# Comparative Technoeconomic Analysis and Life Cycle Assessment of Emerging Reactive Carbon Capture-to-Methanol Pathways

**DOI:** 10.1021/acs.iecr.5c02271

**Published:** 2025-10-15

**Authors:** Jonathan A. Martin, Eric C. D. Tan, Daniel A. Ruddy, Anh T. To

**Affiliations:** 53405 National Renewable Energy Laboratory (NREL), 15013 Denver West Parkway, Golden, Colorado 80401, United States

## Abstract

Our group recently developed dual-function materials (DFMs) and reactive carbon capture (RCC) processes for the selective production of methanol (MeOH) or CO, offering two novel and unique pathways for MeOH production. This study conducted a comparative techno-economic analysis (TEA) of the two RCC pathways from exhaust CO_2_: 1) a “Direct RCC-to-MeOH” pathway and 2) an “Indirect RCC-to-CO” pathway followed by MeOH synthesis. The “Direct RCC-to-MeOH” pathway produced a lower levelized cost of MeOH (LCOM) at $0.78/kg, compared to $0.84/kg for the “Indirect RCC-to-CO” pathway. The key difference is the need to recompress the syngas from RCC before MeOH synthesis in “Indirect RCC-to-CO.” Nonetheless, with reduced catalyst costs and hydrogen requirements for “RCC-to-CO,” this pathway merits further study to produce syngas rather than MeOH. Both pathways are comparable in LCOM to baseline e-MeOH production from CO_2_ hydrogenation ($0.72/kg) while having lower carbon intensities (0.45 and 0.51 kg-CO_2_e/kg vs 0.54 kg-CO_2_e/kg).

## Graphical abstract

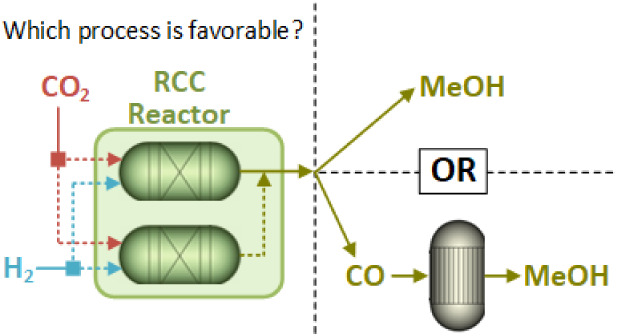

## Introduction

Research into nonpetroleum alternatives for methanol (MeOH) production has seen significant interest in recent years.^1–4^ MeOH can be used both directly in marine transport^5,6^ and as a precursor for sustainable aviation fuel.^7^ Currently, both biomass- and electricity-based MeOH (“e-MeOH”) production routes have yet to demonstrate cost competitiveness with fossil-derived MeOH, though they could potentially become competitive as these technologies mature.^2^ There are many potential routes to yield MeOH, ranging from the conventional steam methane reforming/methanol synthesis pathway to several electrolysis-based pathways,^8^ including those that isolate CO_2_ from waste streams.^9^ Even though much progress has been made in the production of MeOH from CO_2_ hydrogenation,^10–13^ the energy and capital investment required to generate, purify, and transport pure CO_2_ streams to utilization facilities introduce significant cost drivers to the products from CO_2_ hydrogenation, thereby impeding widespread implementation of these technologies. Additionally, CO_2_ transportation would introduce further barriers to scaling CO_2_ utilization technologies due to the lack of infrastructure and the permitting challenges associated with building such infrastructures.^14^

Therefore, the development of technologies that efficiently convert waste CO_2_ from dilute sources (such as power plant exhausts) into a value-added product, such as e-MeOH, without the need for intermediate CO_2_ separation, purification, and transport steps, is important for strengthening economic resilience, promoting workforce development, and expanding fuel and chemical supply chains.^15^ On this front, reactive carbon capture (RCC) has been demonstrated as a promising technology for e-MeOH production by reducing costs through process intensification. The RCC technology features a two-step process carried out in a single reactor, in which CO_2_ from a dilute source is both captured and reacted via a uniquely developed dual-function material (DFM). In the first step, dilute CO_2_ flows into the reactor over a DFM, and CO_2_ is captured by the DFM via the formation of surface intermediates. In the second step, the same reactor is heated to a desired temperature in an atmosphere of hydrogen to trigger the reactive desorption of the captured CO_2_ to form targeted products. This process was first described in a study by Martin et al.,^16^ which developed a framework to perform combined technoeconomic analysis (TEA) and life cycle assessment (LCA). At the end of this initial study, it was found that an unmodified copper–zinc–alumina (CZA) catalyst, which is a commercial MeOH synthesis catalyst, was insufficient to reduce costs over the baseline e-MeOH production via separated CO_2_ capture and conversion technology, due to the lack of selectivity in the products during reactive desorption and the need to conduct several iterations of recycling the tail gases to achieve sufficient purity of MeOH.

From here, our laboratory explored two simultaneous paths for DFM development. In the first, the CZA catalyst was modified via deposition of alkali/alkaline earth metal oxides to facilitate both enhanced CO_2_ capture performance and MeOH selectivity during reactive desorption.^17^ Modification of CZA with a Group I metal oxide, specifically K_2_O forming K/CZA DFM, resulted in the greatest promotional effect, with the highest MeOH selectivity (46%) and a 4.5x increase in MeOH productivity compared to unmodified CZA. In the second path, similar work was performed but with metallic-phase-free zinc–alumina mixed oxides being modified with K, in order to target high selectivity to carbon monoxide (CO) as the product of RCC.^18^ The K modification of equimolar Zn:Al mixed oxides resulted in the best-performing DFM (referred to herein as K/ZA) by balancing surface area for CO_2_ capture and active sites for the hydrogenation reaction, with 97.6% selectivity to CO and a 5x increase in CO productivity compared to the unmodified material. It was hypothesized that this CO could then be combined with excess H_2_ from hydrogenation as a syngas feed for a conventional catalytic MeOH synthesis unit operation. These previously untested catalyst modification techniques offer two separate novel and unique pathways for renewable MeOH production. However, to determine the feasibility of these approaches for e-MeOH production, a comprehensive TEA and LCA study is needed to compare the pros and cons of each pathway with conventional MeOH production methods. Therefore, in this report, we applied the previously developed TEA/LCA framework to the two new pathways using the best-performing DFM for each approach: (1) “Direct RCC-to-MeOH,” featuring a K-modified CZA DFM (“K/CZA”), and (2) “Indirect RCC-to-CO,” featuring a K-modified ZA DFM (“K/ZA”) for syngas production and coupling with conventional MeOH synthesis from syngas, to determine which is optimal for e-MeOH production.

## Methods

As previously described by Martin et al.,^16^ the TEA/LCA framework sends the results of ASPEN Plus modeling for the RCC processes to a Python analysis tool developed by NREL. This tool, HOPP (Hybrids Optimization and Performance Platform),^19^ is integrated into a larger suite of analysis tools called H2Integrate (Holistic Hybrids Optimization and Design Tool).^20^ We will first describe the ASPEN process modeling, followed by an overview of the Python HOPP modeling.

In the ASPEN portion of this framework, the results from laboratory-scale experiments are used to model commercial-scale reactors along with their associated energy and feedstock requirements. The material and energy balance from the process simulation is essential for equipment sizing and costing, as it helps determine the capital costs and estimate operating costs. Both capital and operating costs are key inputs for TEA. Additionally, the material and energy balance provides an inventory for LCA, which is conducted to evaluate environmental impacts. A process model for converting CO_2_ and H_2_ into MeOH was developed for both RCC pathways. The model for the “Direct RCC-to-MeOH” pathway is shown in Figure 1a, while the model for the RCC-to-CO, followed by MeOH synthesis from syngas, i.e., ″Indirect RCC-to-CO,” is illustrated in Figure 1b. The RCC performance of K/CZA and K/ZA, which are the best-performing DFMs for RCC-to-MeOH^17^ and RCC-to-CO,^18^ respectively, as reported previously by our group, were used as inputs for the Aspen modeling.

**Figure 1:**
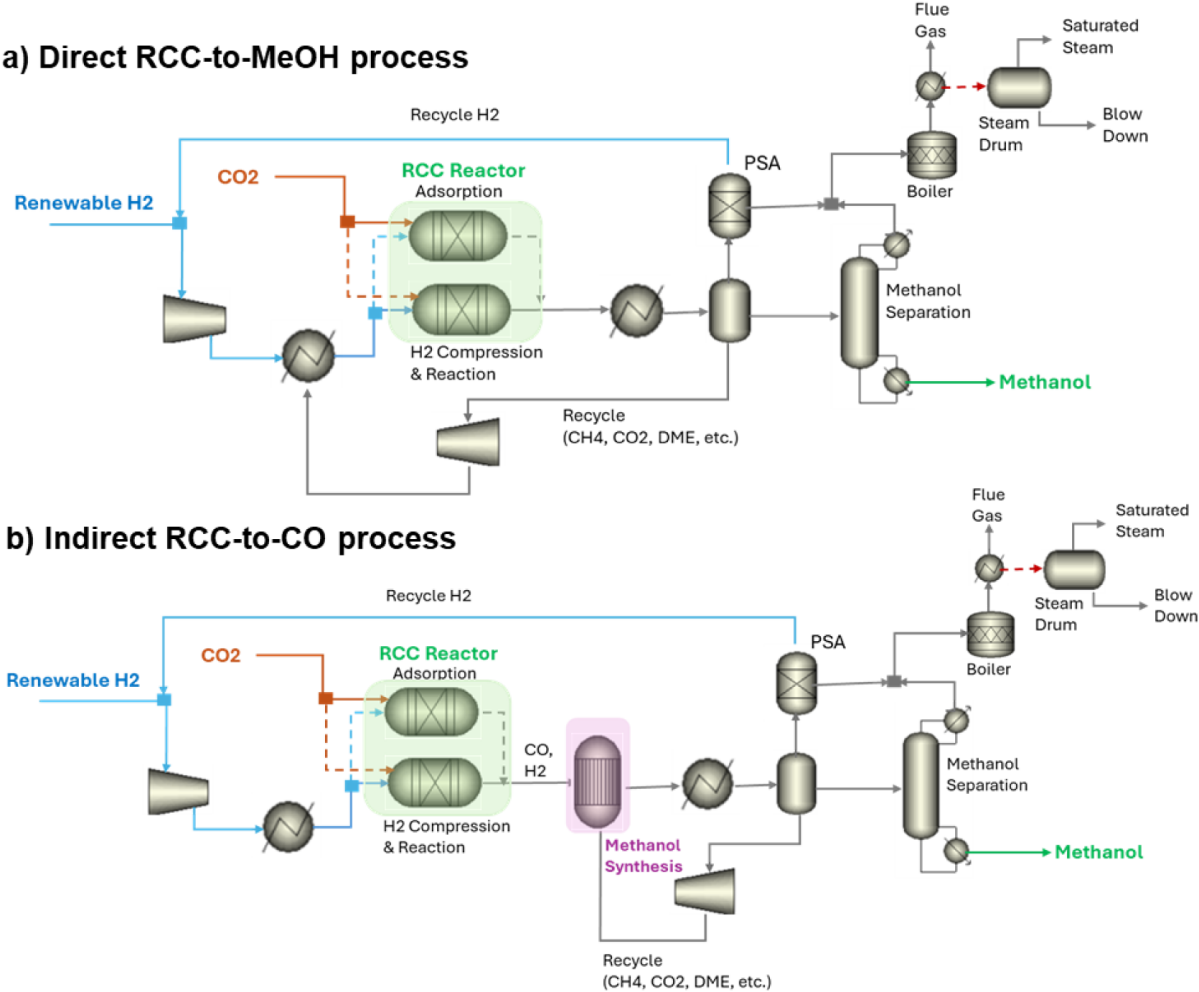
ASPEN Plus process flow diagrams for MeOH production from a) “Direct RCC-to-MeOH” using K/CZA DFM and b) RCC-to-CO followed by MeOH synthesis (“Indirect RCC-to-CO”) using K/ZA DFM.

The process design for Direct RCC-to-MeOH (Figure 1a) is similar to that in Martin et al.,^16^ where the operation of an RCC MeOH production system consists of a two-step process utilizing multiple temperature- and pressure-swing reactors (TPSRs) paired together containing the K/CZA DFM. In the first step, flue gas from the natural gas-fired power plant is introduced into the first TPSR (R1) reactor at low pressure, where carbon dioxide (CO_2_) is adsorbed onto a DFM bed. In the second step, when the DFM bed is saturated with captured CO_2_, the reactor is switched to operate in a reactive desorption mode (as demonstrated in R2), where the reactor undergoes pressurization with hydrogen at 30 bar. During this phase, the temperature in R2 is increased to 250 °C, which facilitates the hydrogenation reaction of adsorbed CO_2_ to form products (CO and MeOH) and regenerates the DFM for the next capture cycle. The DFM performance, including CO_2_ uptake, conversion of captured CO_2_, and MeOH product selectivity, was modeled by using the experimental data summarized in Table 1. Unconverted CO_2_ and coproduct CO are recycled back to the RCC reactor, allowing for additional MeOH production. Excess hydrogen was recovered by using a pressure-swing adsorption (PSA) unit to minimize the need for supplemental hydrogen. Purge gas from the PSA, along with off-gas from MeOH separation, is sent to a boiler for steam generation and process heating. Excess saturated steam is exported for coproduct credit.

**Table 1:** Experimental data used for ASPEN Modeling.^a^

		Capture step	Reactive desorption step								
						Productivity (μmol/g)			Product C-selectivity (mol %)		
Pathway	DFMs	Strong CO_2_ uptake (μmol/g)	Hydrogenation Pressure (bar)	CO_2_ conversion (%)	Desorbed CO_2_ (μmol/g)	MeOH	CO	CH4	MeOH	CO	CH4
**RCC-to-MeOH^a^**	K/CZA	135.3 ± 11.1	30	94.0 ± 3.7	3.6 ± 2.9	59.0 ± 3.5	62.6 ± 0.9	5.6 ± 0.4	46.3	49.3	4.4
**RCC-to-CO^b^**	K/ZA	331.0 ± 41.1	1.01	53.3 ± 0.5	182 ± 6.5	0	202 ± 3.7	4.9 ± 1.7	0.0	97.6	2.4

a Data From Jeong-Potter et al.^17^

b Data From Hill et al.^18^

Even though having a similar operational principle, the process design for “Indirect RCC-to-CO” (Figure 1b) differs from “Direct RCC-to-MeOH.” In the former case, reactive desorption takes place at a lower pressure of 1.01 bar and a higher temperature of 400 °C, while the latter process operates at a pressure of 30 bar and 250 °C. Additionally, the primary product of the RCC-to-CO process is CO with a selectivity of 97.6%, whereas the RCC-to-MeOH process yields a mixture of MeOH and CO (Table 1). The RCC effluent, which contains CO and H_2_, is compressed to 50 bar for MeOH synthesis. This process utilizes a commercial CZA catalyst in a fixed-bed reactor.^21^ One major concern is the heat released during the reaction, as even small temperature increases of just a few degrees can irreversibly damage the catalyst. To manage this, heat must be removed from the reactors since the synthesis reaction is exothermic. Temperature control is achieved by generating steam on the shell side of the reactor. The unconverted products and reactants from MeOH synthesis (CO_2_ and CO) are recycled to the MeOH synthesis reactor, instead of the RCC reactor as in the “Direct RCC-to-MeOH” process, whereas H_2_ recovery and recycling are similar between the two processes.

The results of this ASPEN modeling (as shown in Table S3) are then used in H2Integrate (abbreviated as H2I) to determine a full “cradle-to-gate” model of all the inputs required for the complete e-MeOH production process, including sourcing CO_2_ from industrial flue gas and hybrid wind/solar/grid electricity for powering H_2_ production. The H_2_ is produced by proton exchange membrane (PEM) electrolysis, powered by a hybrid wind/solar electric plant optimized for the location (central TX). This plant is grid-connected, with the cumulative wind and solar production matching or exceeding the cumulative load of the electrolyzer, and the exact size of the wind and solar assets determined by an optimization routine. The exact price and emission factor for hydrogen will depend on the location and size of the plant due to wind/solar resource availability and economies of scale (see Martin et al.^16^ for details). Figure 2 shows the full framework, with H2I receiving “performance” inputs (e.g., mass balances) and “cost” inputs (e.g., capital and operating costs) from ASPEN (Table S3) to model the RCC system. H2I then calculates a full system design, including a flue gas source (i.e., flue gas from a natural gas-fired power plant) and an electrolyzer, which will provide CO_2_ and H_2_ at the rates necessary for a specified production rate of MeOH (ca. 115 k tonne/yr). H2I can also modify H_2_ costs and emissions to simulate MeOH production costs and emissions with more conventional H_2_ sources. In all cases, renewable electricity (from the same wind-solar hybrid used to power renewable H_2_ production) is used to meet the energy needs of the RCC reactor. The total net emissions of CO_2_-equivalent gases (abbreviated as CO_2_e) and consumption of water are used to calculate the carbon intensity (CI) and water consumption (WC), respectively. Meanwhile, the full capital and operating costs of the system are sent to a financial model that calculates a levelized cost of MeOH (LCOM) over the plant lifetime. This includes costs for modifying the base CZA and ZA catalysts through incipient wetness impregnation (IWI), which were estimated using the CatCost tool from ChemCatBio^22^ and shown in Table 2. Further details on this framework can be found in Martin et al.,^16^ which includes details of the system boundaries and data sources that feed into H2Integrate.

**Figure 2:**
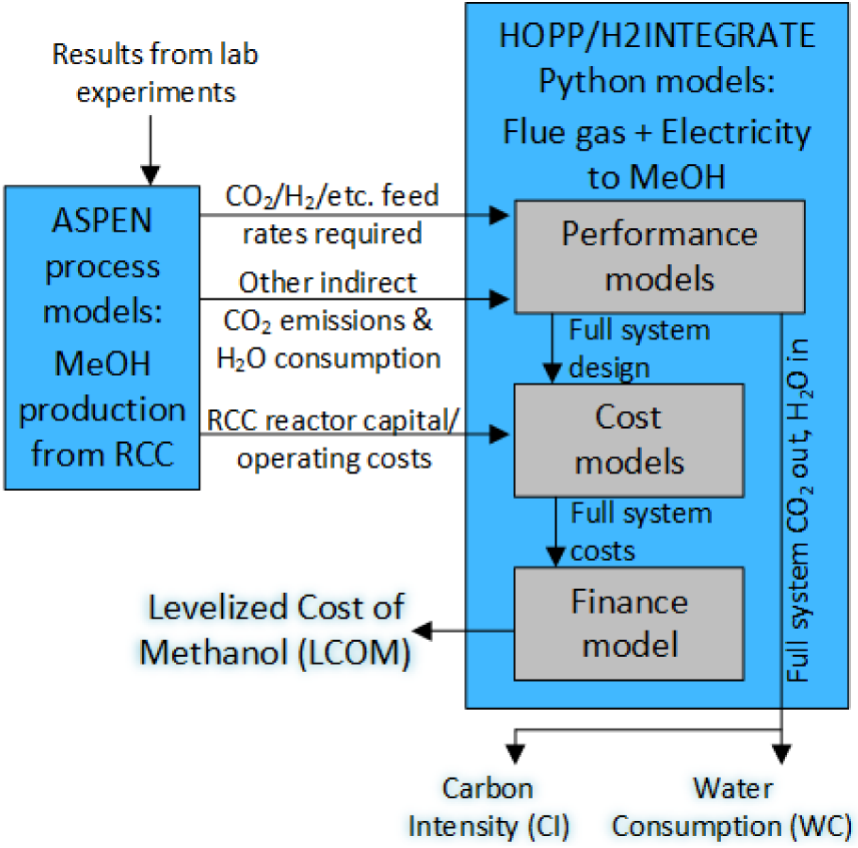
A schematic of the entire TEA/LCA framework shows the use of experimental data to feed ASPEN process modeling of the standalone RCC process, which was then used to feed the larger HOPP/H2Integrate model of the entire “cradle-to-gate” MeOH system, starting with flue gas (used to extract CO_2_) and water (used to produce H_2_).

**Table 2:** Cost Estimates for Bulk Manufacture of DFM for RCC Using CatCost Based on Incipient Wetness Impregnation (IWI) of Commercial Mixed-Metal Oxide Supports

DFM	Commercial base material cost ($/tonne)	IWI total process cost, including precursor materials ($/tonne)	Net final catalyst cost ($/tonne)
K/CZA	35,896	9,150	45,046
K/ZA	28,288	9,150	37,438

In addition to the RCC systems, we also perform comparative TEA/LCA of two baseline MeOH production systems: (1) conventional steam methane reforming (SMR) using natural gas (NG) to produce MeOH, and (2) CO_2_ hydrogenation to produce MeOH from H_2_ and a pure CO_2_ stream captured from flue gas. These systems are not based on any new ASPEN modeling but instead on literature. The SMR system is based on the National Energy Technology Laboratory (NETL) baseline analysis of MeOH production^23^ for the TEA and the NETL life cycle inventory^24^ for the LCA. The CO_2_ hydrogenation system is based on averages from literature TEA/LCA studies of these systems at a comparable scale.^25–27^ In the next section, we will compare the results of the two RCC systems to these two baselines. However, the SMR system will only be used as a baseline when conventional hydrogen prices are used, since SMR itself requires conventional natural gas and is not comparable to CO_2_ hydrogenation or the RCC systems when these systems use renewable electricity to produce hydrogen for MeOH synthesis.

## Results and Discussion

### ASPEN Process Modeling

The results from the process modeling are shown in Table 3, with detailed data for each process unit and stream provided in the Figures S1, S2 and Tables S1, S2). The first key takeaway from this modeling is that capital costs are much higher for the “Indirect RCC-to-CO” process than for the “Direct RCC-to-MeOH” process ($41.41 MM versus $24.22 MM, respectively). This is due to the additional process equipment—specifically the MeOH synthesis reactor—required for the “Indirect RCC-to-CO” approach, which includes an extra step of MeOH synthesis following RCC. Other disadvantages of “Indirect RCC-to-CO” are the greater usage of electricity (8.5x) and process water (4.1x). Large amounts of electricity are needed in the “Indirect RCC-to-CO” system to compress the syngas (CO + H_2_) mixture coming from RCC (which occurs at atmospheric pressure) to the high pressure required for MeOH synthesis (50 bar). Meanwhile, more process water is needed to largely supply the cooling load for the “Indirect RCC-to-CO” process due to the recompression of the RCC effluent for the MeOH reactor, which produces excess heat, and an overall increase in evaporative water loss from the cooling tower.

**Table 3:** ASPEN Modeling Results

	Process	RCC-to-MeOH	RCC-to-CO
**Process model inputs**	Catalyst	K/CZA	K/ZA
	Catalyst replacement cycle (years)	3	3
	Flue gas CO_2_ feed (tonne/yr)	871,546	949,289
	MeOH production (tonne/yr)	115,104	115,104
	Plant capacity factor (% time operating)	90%	90%
	Net methanol output during operation (kg/h)	14,600	14,600
	Hydrogenation pressure (bar)	30	1.01
**Process performance output**	MeOH yield (tonne MeOH/tonne CO_2_)	0.13	0.12
	RCC catalyst required (tonnes)	930	413
	MeOH synthesis catalyst required (tonnes)	0	54
	Hydrogen usage during operation (kg/h)	3,695	3,270
	H_2_ consumption (tonne H_2_/tonne MeOH)	0.25	0.22
	Electricity usage during operation (kW)	7,151	61,258
	Net process water usage during operation (kg/h)	40,586	167,513
	Liquid product methanol purity (wt %)	95.6%	95.3%
**Process cost output**	Total installed cost (mil. 2020 $)	$12.23	$21.33
	Fixed capital investment excl. land (mil. 2020 $)	$23.02	$39.43
	Working capital (mil. 2020 $)	$1.15	$1.97
	Total capital investment (mil. 2020 $)	$24.22	$41.41

However, the “Indirect RCC-to-CO” system has two key advantages over the “Direct RCC-to-MeOH” system: first, “Indirect RCC-to-CO” requires less than half the amount of DFM, and second, it has higher H_2_ efficiency compared to “Direct RCC-to-MeOH,” as indicated by lower H_2_ per unit of MeOH produced (ca. 10% less). The higher CO_2_ capture capacity on K/ZA (331 μmol_CO2_/g_DFM_) compared to K/CZA (135 μmol_CO2_/g_DFM_) mainly accounts for the lower DFM demand in the “Indirect RCC-to-CO” process. In the Aspen model, the amount of hydrogen required during the reactive desorption step remains constant, as it is determined by a fixed input H_2_/CO_2_ ratio. However, the productivity and selectivity of the process can affect the demand for hydrogen in MeOH synthesis, while the input H_2_/CO_2_ ratio remains unchanged. Specifically, the H_2_ efficiency of the process decreases with the formation of undesired product (i.e., CH_4_), as it cannot be converted to MeOH in the MeOH synthesis reactor or during product recycling. The “Indirect RCC-to-CO” process has lower selectivity to CH_4_ (2.4%) compared to “Direct RCC-to-MeOH” (4.4%) and therefore has higher H_2_ efficiency.

### H2Integrate Modeling

The results of the TEA in H2I are shown in Figure 3, for processing using both (a) renewable hydrogen (via PEM electrolysis) and (b) conventional fossil-derived precursors (i.e., natural gas and hydrogen). The three renewable routes (Figure 3a) are all roughly comparable in price, and for all three, hydrogen is by far the largest cost component (accounting for 69 – 82% of total LCOM). Although neither RCC process uses hydrogen as efficiently as CO_2_ hydrogenation, the “Direct RCC-to-MeOH” process has the lowest non-hydrogen costs, of which the catalyst cost is the second-largest component after H_2_ cost (16% of total LCOM). Due to imperfect selectivity, “Direct RCC-to-MeOH” also produces combustible tail gases, which can generate steam for credit. The CO_2_ generated by this combustion is accounted for in the LCA. As indicated above, although “Indirect RCC-to-CO” uses hydrogen more efficiently due to better catalyst selectivity for CO and lower selectivity to CH_4_, it cannot produce these same steam credits and has a large cost for the electricity needed for syngas compression (“Reactor electricity bar,” $0.16/kg). As a result, the “Indirect RCC-to-CO” process has a higher LCOM ($0.84/kg) than the ″Direct RCC-to-MeOH” process ($0.78/kg). These RCC processes still have slightly higher LCOM than the baseline CO_2_ hydrogenation process using conventional separated CO_2_ capture and conversion technology ($0.72/kg). With the conventional precursors (Figure 3b), hydrogen costs (at $1.50/kg H_2_) are much lower for the three processes that utilize hydrogen, but the LCOM is still significantly higher than the baseline SMR process (2.2–2.6 times higher), which uses only natural gas.

**Figure 3:**
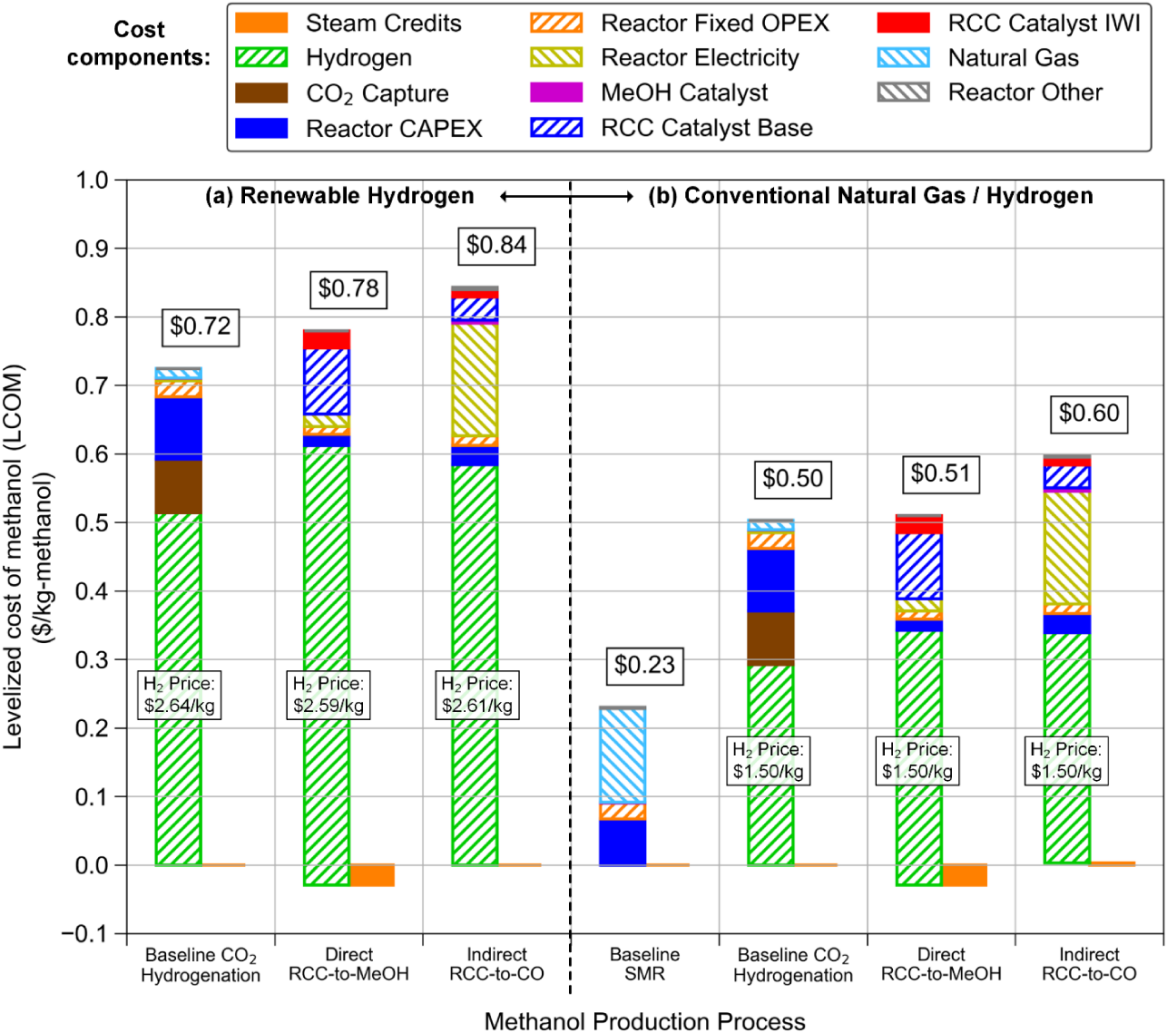
TEA results for (a) processes utilizing renewable hydrogen and (b) processes utilizing either conventional natural gas or hydrogen (which, in turn, is produced from natural gas) as the main feedstock for the production of MeOH.

It is important to consider the sensitivity of the LCOM to hydrogen cost, since hydrogen is the largest cost component. The RCC processes are more sensitive to hydrogen cost than the baseline CO_2_ hydrogenation process—a change of $1.00/kg-H_2_ in the hydrogen cost will lead to a change of $0.20/kg-MeOH in the LCOM of CO_2_ hydrogenation, but $0.25/kg-MeOH in RCC-to-MeOH and $0.22/kg-MeOH in RCC-to-CO. The cost of hydrogen from electrolysis is itself largely dependent on the cost of electricity. In this modeling, a low levelized cost of electricity (LCOE) of $33.2/MWh was found for an ideal mix of wind and solar electricity in central Texas, which enables the hydrogen prices used in the TEA calculation (Figure 3). Using the current industrial electricity price for that location of $63.4/MWh^28^ would increase the price of CO_2_ hydrogenation by $0.32/kg-MeOH, while increasing the price of RCC-to-MeOH by $0.41/kg-MeOH and RCC-to-CO by $0.37/kg-MeOH, because of their varying hydrogen consumption ratios.

In contrast to the TEA, the LCA shows improved carbon intensity (CI) for both RCC processes compared to the baseline CO_2_ hydrogenation process when using renewable hydrogen (Figure 4a). For all three processes, hydrogen production accounted for the majority of the carbon emissions (59–90% of total CI). Even though renewable electricity is used for hydrogen production from water electrolysis, the hydrogen quantities required are such that the CO_2_ emissions become significant in terms of kg-CO_2_e/kg-H_2_, even though they are relatively low in terms of kg-CO_2_e/kWh for the electricity supplied to electrolysis. Despite having better hydrogen efficiency, the “Indirect RCC-to-CO” pathway has a higher CI (0.51) than the “Direct RCC-to-MeOH” pathway (0.45) due to the additional electricity requirement for syngas compression, as previously discussed in the TEA. The baseline CO_2_ hydrogenation approach has the highest CI (0.54), regardless of its lower hydrogen usage, because the intermediate CO_2_ desorption, purification, and compression steps prior to CO_2_ utilization create an additional burden of indirect CO_2_ emissions (CO_2_ capture CI of 0.15 kg-CO_2_e/kg-MeOH in Figure 4a). When a conventional hydrogen source is used, the CI for MeOH production from CO_2_, either via RCC processes or separated CO_2_ capture and conversion (i.e., baseline CO_2_ hydrogenation), increases significantly (1.61–1.82) and is much higher than that of the conventional MeOH production technology (i.e., baseline SMR, CI of 1.13). This is expected, because MeOH production from CO_2_ hydrogenation requires a lot more hydrogen than production from syngas (CO_2_ hydrogenation to MeOH consumes 3 mol of H_2_, while MeOH synthesis from syngas requires 2 mol of H_2_), resulting in substantially higher CI from conventional hydrogen generation. Hydrogen efficiency thus becomes the most important factor determining the CI intensity of the processes, i.e., the CI intensity follows the order of H_2_ consumption of the processes: baseline CO_2_ hydrogenation < “Indirect RCC-to-CO” < “Direct RCC-to-MeOH.”

**Figure 4:**
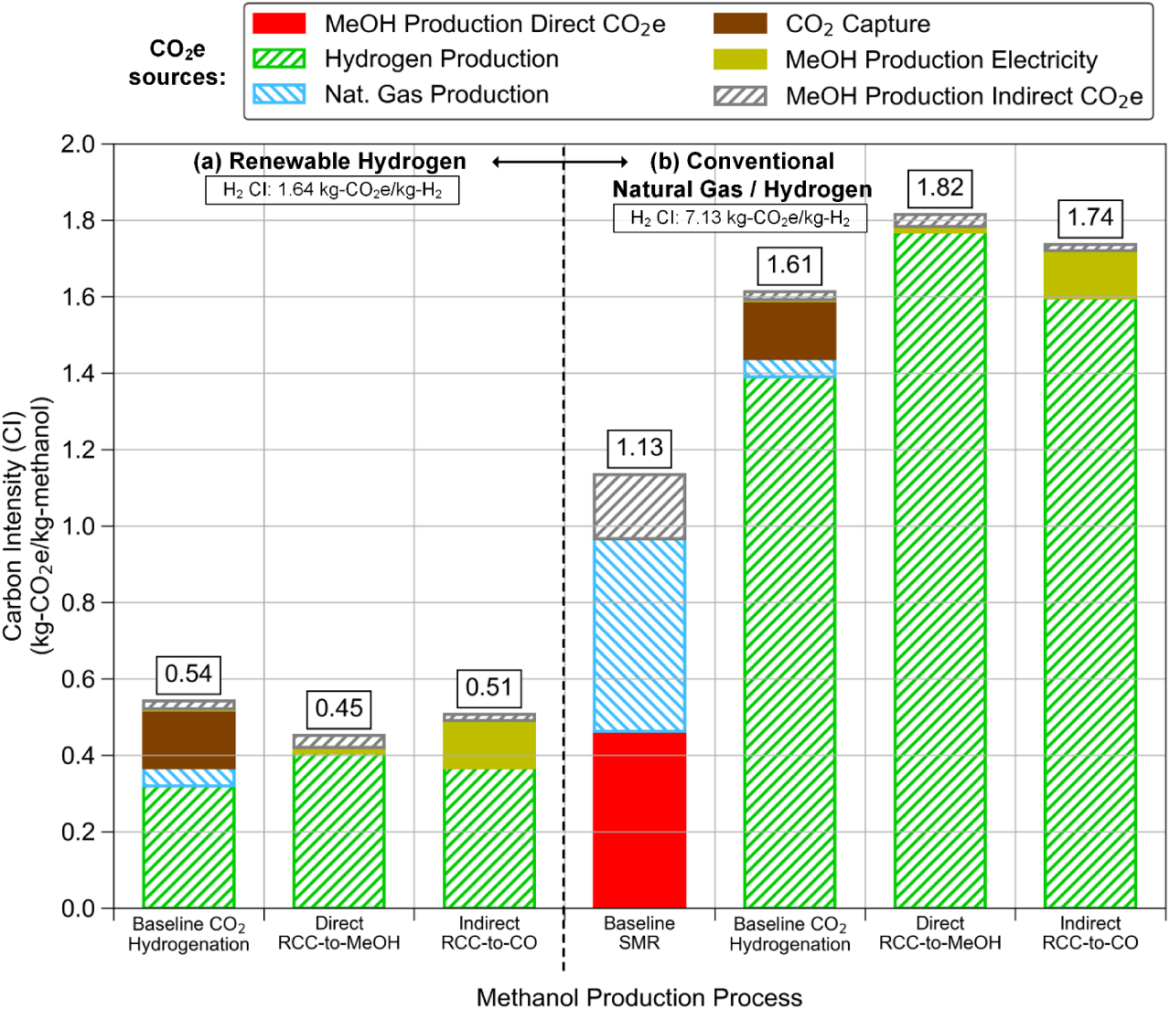
LCA Carbon Intensity (CI) results for (a) processes utilizing renewable hydrogen and (b) processes utilizing either conventional natural gas or hydrogen (which is, in turn, produced from natural gas) as the main feedstock for the production of MeOH.

Similar to the TEA, the CI of the MeOH production processes is highly sensitive to the CI of hydrogen production, which is, in turn, highly sensitive to the CI of the electricity used to produce the hydrogen. The wind/solar mix (dominated by wind) from central Texas used here is again ideal, achieving an electricity carbon intensity of only 0.028 kg-CO_2_e/kWh according to the NETL Grid Mix Explorer,^29^ which leads to the low CI for hydrogen production of 1.64 kg-CO_2_e/kg-H_2_. However, this could be a conservative estimate, since a median value from other studies places the CI of wind power as low as 0.012 kg-CO_2_e/kWh.^30^ With electricity CI this low, the hydrogen CI would be significantly lower at 0.76 kg-CO_2_e/kg-H_2_, dropping CO_2_ hydrogenation CI to 0.37 kg-CO_2_e/kg-MeOH, RCC-to-MeOH to 0.23 kg-CO_2_e/kg-MeOH, and RCC-to-CO to 0.30 kg-CO_2_e/kg-MeOH. Conversely, one study found wind power carbon intensity to be as high as 0.081 kg-CO_2_e/kWh,^30^ which would place the CI of all 3 renewable routes within 10% of the baseline SMR route. This highlights how the entire production pathway, and all potential direct and indirect carbon emissions, must be fully considered to determine the carbon impact of the fuel whenever renewable electricity is used to generate fuel.

Turning to the water consumption (WC) portion of the LCA, the “Direct RCC-to-MeOH” process clearly had the lowest WC of any process using renewable hydrogen, as shown in Figure 5a at 6.1 kg_H2O_/kg_MeOH_. Similar to CI, the WC advantage of the “Direct RCC-to-MeOH” process resulted from the avoidance of the intermediate CO_2_ desorption, purification, and compression steps prior to CO_2_ utilization. In the baseline CO_2_ hydrogenation approach, CO_2_ is captured by an amine solvent, followed by CO_2_ desorption and solvent regeneration using steam, which requires large WC. Additionally, such a CO_2_ capture system greatly increases the need for cooling water through the plant’s cooling tower, increasing evaporative losses and thus net WC.^31^ These large WC during the CO_2_ adsorption–desorption step are indicated as the brown bar in Figure 5 (9.2 kg_H2O_/kg_MeOH_). Even though the “Indirect RCC-to-CO” process can avoid the intermediate CO_2_ desorption step, which has large WC, the need for cooling water to recompress the RCC effluent for MeOH synthesis contributed to much higher WC (MeOH reactor bars in Figure 5a, 11.5 kg_H2O_/kg_MeOH_). Using conventional hydrogen instead of renewable hydrogen does not have a large impact on the overall WC; however, as seen in Figure 5b, the baseline SMR technology has a much lower WC than any other pathway.

**Figure 5:**
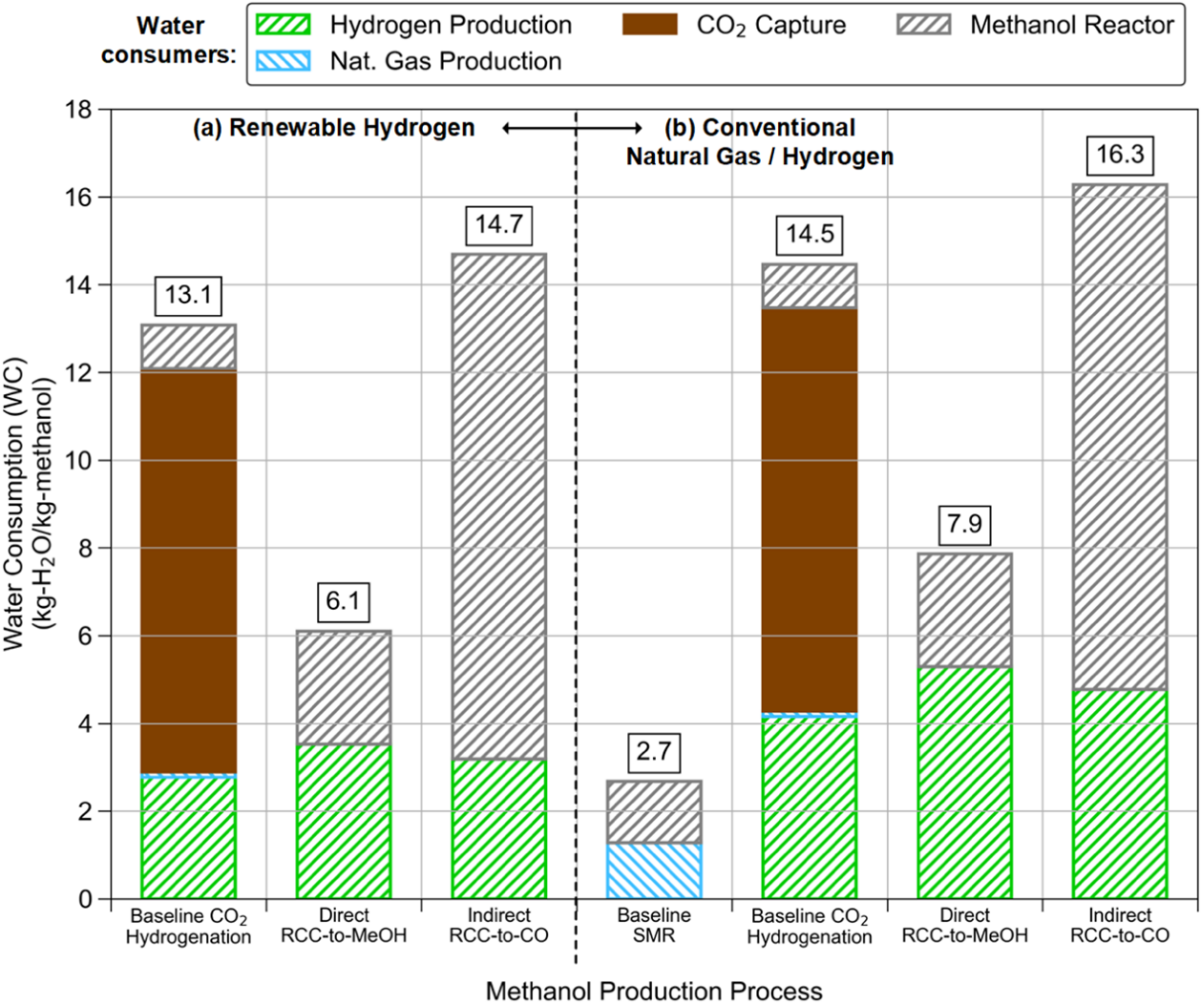
LCA Water Consumption (WC) results for (a) processes utilizing renewable hydrogen and (b) processes utilizing either conventional natural gas or hydrogen (which is, in turn, produced from natural gas) as the main feedstock for the production of MeOH.

### Relative Costs and Sensitivity Scenarios

Based on all the information presented in the previous sections, it is essential to compile the most important factors into a single diagram to provide a comprehensive comparison of MeOH production from the “Direct RCC-to-MeOH” and “Indirect RCC-to-CO” processes. This is illustrated in Figure 6, which plots five of the most important metrics for both processes on a radar or “spider plot,” where the data points closer to the edge of the pentagon show greater advantages of the process. The “Direct RCC-to-MeOH” process demonstrates considerable advantages regarding LCOM and WC, along with a minor advantage in CI. However, the “Indirect RCC-to-CO” process also has advantages, as it requires significantly less hydrogen and considerably less DFM to produce the same amount of MeOH as the “Direct RCC-to-MeOH” process. The reason these advantages do not lead to benefits for the “Indirect RCC-to-CO” process in the overall TEA/LCA is attributed to the ambient-pressure operation for the reactive desorption, which results in substantial costs and cooling losses from recompression of the resulting syngas from the RCC unit for subsequent MeOH synthesis. This analysis provides guidance for future study, which is to understand the effect of intermediate reactive desorption pressures, such as 10–20 bar, on CO per-cycle yield and selectivity of CO and the resulting effect on the TEA/LCA results.

**Figure 6:**
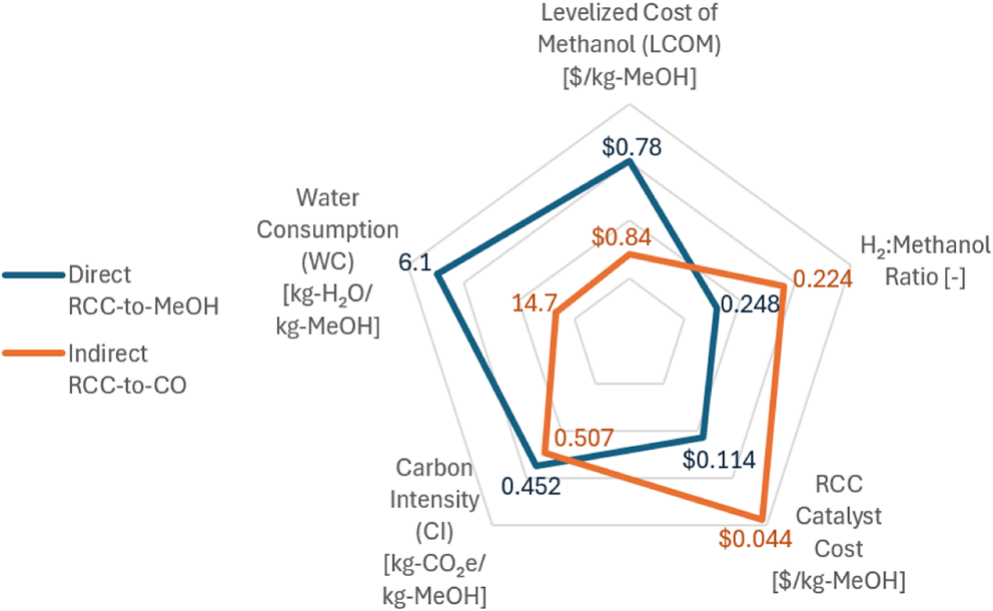
Radar plot shows the relative advantages of the ″Direct RCC-to-MeOH” and “Indirect RCC-to-CO” pathways. For all five axes, lower values are preferred, so they are plotted further out on the axis. The ″Direct RCC-to-MeOH” pathway has lower LCOM and WC, whereas “Indirect RCC-to-CO” has lower H_2_:MeOH ratio and catalyst mass requirements, while their CI is approximately the same.

Another consideration for the RCC-to-CO pathway is that MeOH is not the only possible product of this pathway. The syngas resulting from RCC could be sold as fuel or as a chemical intermediate itself without being further upgraded to MeOH. Since the original purpose of this study was to explore pathways to produce MeOH, the TEA/LCA did not consider the costs or emissions of standalone syngas production, but this would be an interesting topic for future work.

Regardless of the pathway, current limitations in renewable hydrogen production will limit the economic competitiveness of e-MeOH. It is worth, however, considering future scenarios in which renewable hydrogen technology develops to reduce the cost of hydrogen production and/or natural gas becomes more expensive. Figure 7 shows two scenarios, both of which involve significant technological development to reduce renewable hydrogen production costs to $1.50/kg. In both cases, the oxygen that is coproduced with hydrogen during water electrolysis is sold as a coproduct for a credit of $0.05/kg. In Figure 7a, natural gas is kept at its current cost of $4.00/MMBtu, in which case all the e-MeOH pathways are much more expensive than SMR. However, as shown in Figure 7b, if natural gas costs reach $10.00/MMBtu (as they have at peaks in the past),^32^ then the RCC-to-MeOH pathway would have a lower cost than both SMR and CO_2_ hydrogenation. Although this analysis represents only a projected future scenario, it demonstrates the pathway by which RCC could produce MeOH at a lower cost than all baseline technologies.

**Figure 7:**
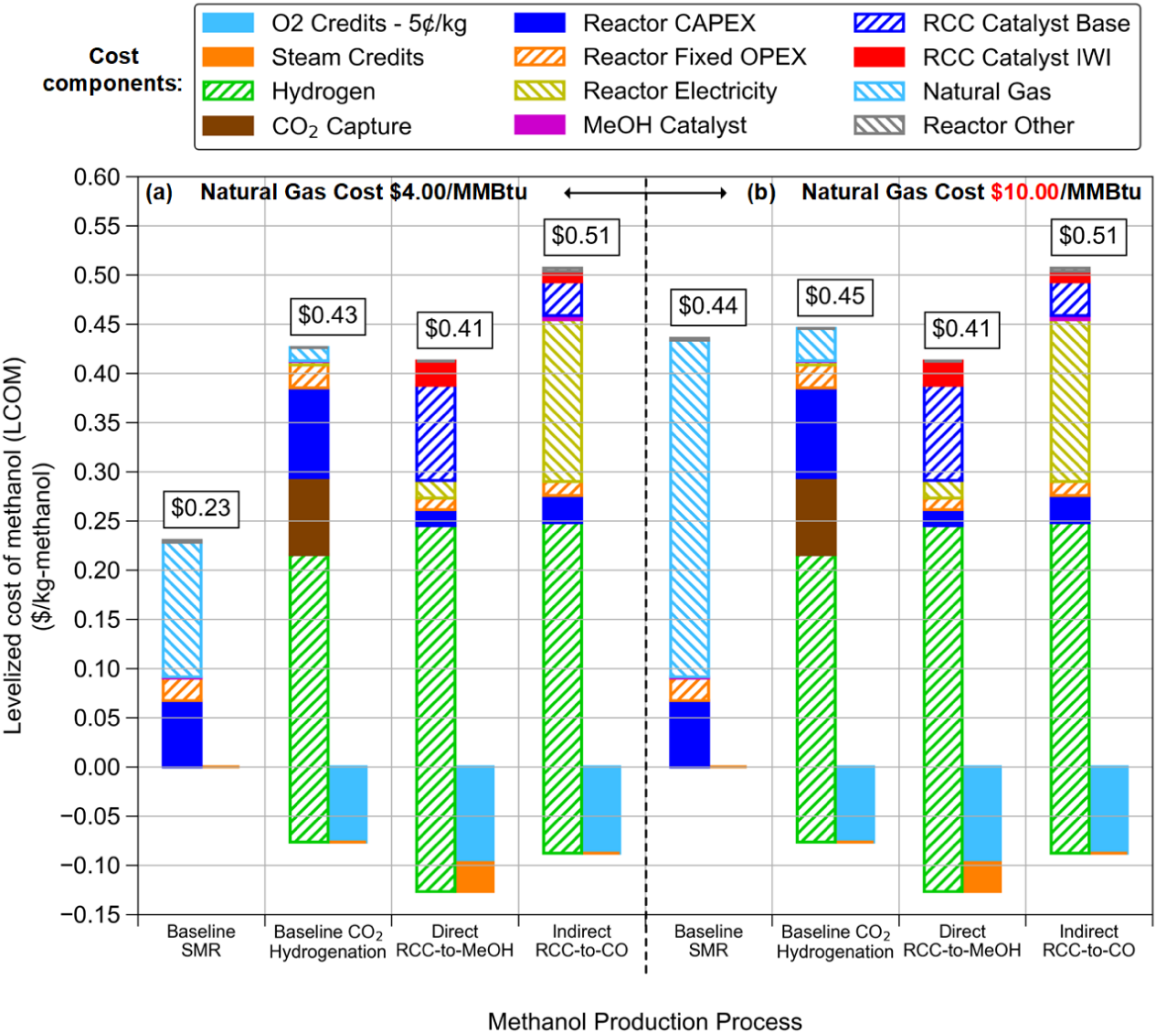
Pathway to economic competitiveness for MeOH production from RCC vs baseline SMR and CO_2_ hydrogenation processes. (a) With a future renewable H_2_ cost of $1.50/kg, “Direct RCC-to-MeOH” could be competitive with CO_2_ hydrogenation, especially if the oxygen coproduced with renewable hydrogen can be sold as a coproduct. (b) For “Direct RCC-to-MeOH” to be competitive with conventional MeOH production from SMR, in addition to low H_2_ costs, natural gas prices would need to be close to their historical peak, i.e., $10.00/MMBtu.

## Conclusions

This study provides comprehensive TEA and LCA comparisons of two emerging RCC pathways to determine the feasibility of these approaches for e-MeOH production. Of these two potential RCC pathways, “Direct RCC-to-MeOH” shows the clearest potential for economic and environmental benefits. According to the TEA scale-up of current experimental results, this pathway can be used to produce MeOH with an LCOM of $0.78/kg, very close to the current baseline of $0.72/kg for the current state-of-the-art separate CO_2_ capture and hydrogenation approach. In addition, the “Direct RCC-to-MeOH” approach reduce CI and WC (0.45 kg-CO_2_e/kg-MeOH and 6.1 kg-H_2_O/kg-MeOH, respectively) compared to the baseline technology (0.54 kg-CO_2_e/kg-MeOH and 13.1 kg-H_2_O/kg-MeOH, respectively). Experimental optimization of the DFM and RCC reaction conditions could bring these costs below the baseline, and work is currently underway to investigate this possibility. The “Indirect RCC-to-CO” pathway does not show the same benefits, despite having significant advantages over “Direct RCC-to-MeOH” by lowering DFM cost ($0.044/kg-MeOH vs $0.114/kg-MeOH) and improving H_2_ efficiency (H_2_:MeOH ratio of 0.224 vs 0.248). These advantages were canceled out by the process requirements of MeOH synthesis in the “Indirect RCC-to-CO” process. For this reason, it would be worthwhile to investigate RCC-to-CO as a direct syngas production process in further work, rather than as an indirect MeOH production process.

## Supplementary Material



## Notes

Stream tables and process flow diagrams for ASPEN models and detailed TEA results (PDF)

## Abbreviations

RCC: Reactive carbon capture
DFM: Dual-function material
MeOH: Methanol
CO_2_: Carbon dioxide
CO: Carbon monoxide
TEA: Technoeconomic analysis
LCA: Life cycle assement
CZA: Copper–zinc–alumina mixed oxides
IWI: Incipient wetness impregnation
HOPP: Hybrids Optimization and Performance Platform
H2Integrate: Holistic Hybrids Optimization and Design Tool
TPSR: Temperature and pressure swing reactors
PSA: Pressure swing adsorption
LCOM: Levelized cost of methanol
CI: Carbon intensity
WC: Water consumption
SMR: Steam methane reforming
NG: Natural gas
MMBtu: Million British thermal units
