# Supplementary material for: Comparative Technoeconomic Analysis and Life Cycle Assessment of Emerging Reactive Carbon Capture-to-Methanol Pathways
Source: Ind Eng Chem Res. 2025 Oct 15;64(43):20525–34. doi: 10.1021/acs.iecr.5c02271 (PMC12576764; doi:10.1021/acs.iecr.5c02271)
Supplement: Supplementary file 1 [file ie5c02271_si_001.pdf]

Supporting Information

Paper Title: "Comparative Techno-Economic Analyses and Life Cycle Assessment for Emerging Reactive Carbon Capture to Methanol Pathways"

Authors: Jonathan A. Martin[1]*, Eric C. D. Tan[1], Daniel A. Ruddy[1], Anh T. To[1*]

[1]National Renewable Energy Laboratory (NREL), 15013 Denver West Parkway, Golden, CO 80401, USA

*Corresponding author emails: jonathan.martin@nrel.gov, anh.to@nrel.gov

Number of Pages: 3

Figures:

Figure S-1. ASPEN process flow diagrams for MeOH production from "Direct RCC-to-MeOH" using K/CZA DFM.

Figure S-2. ASPEN process flow diagrams for MeOH production from RCC-to-CO followed by MeOH synthesis ("Indirect RCC-to-CO") using K/ZA DFM.

Tables:

Table S-1. ASPEN model stream table for the "Direct RCC-to-MeOH" case (as shown in Figure S-1)

Table S-2. ASPEN model stream table for the "Indirect RCC-to-CO" case (as shown in Figure S-2)

Table S-3. ASPEN model outputs used in TEA calculations

Table S-4. Levelized Cost of Methanol (LCOM) calculations using equation (1)

Table S-5. Calculating contribution to the Variable operating cost (VOC) from electricity, hydrogen, and carbon dioxide

Table S-6. Calculating total Variable operating cost (VOC) from all components

SUPPORTING INFORMATION

All monetary values are in real terms, 2022 dollars with the Chemical Engineering Plant Cost Index (CEPCI) used to correct process equipment capex costs and the consumer price index (CPI) used to correct all other costs reported by other sources in other years before 2022.

ASPEN Modeling

Individual stream tables are given in Table S-1 and Table S-2. Final results which were fed into the HOPP/H2I modeling framework are given in Table S-3.

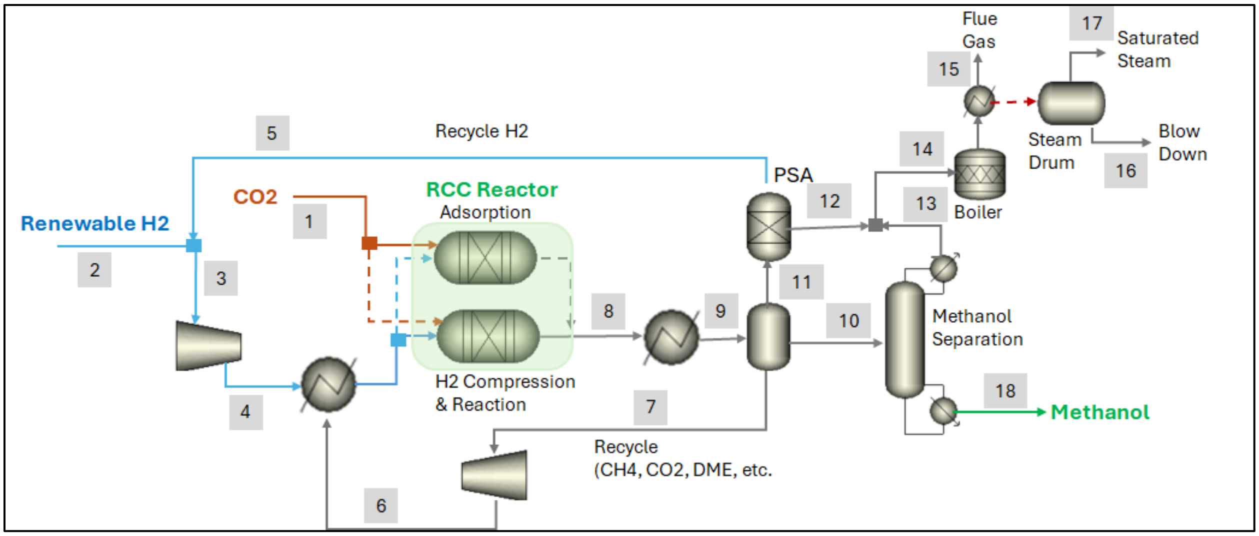

Figure S-1. ASPEN process flow diagram for MeOH production from "Direct RCC-to-MeOH" using K/CZA DFM.

Table S-1. ASPEN model stream table for the "Direct RCC-to-MeOH" case (as shown in Figure S-1)

Stream #	1	2	3	4	5	6	7	8	9
Temperature, °F	60.0	60.0	92.1	199.0	110.0	223.6	121.4	482.0	200.0
Pressure, psia	14.7	350.0	272.0	435.0	272.0	440.0	267.0	435.0	279.0
Mass flow, lb/hr									
CO2	220462.3	-	-	-	-	16470.3	16470.3	17630.3	17630.2
CO	-	-	-	-	-	26693.1	26693.1	28144.3	28142.8
H2	-	7227.8	20196.7	20196.7	12968.8	27507.8	27507.8	41929.6	41929.6
H2O	-	-	-	-	-	1075.7	1075.7	19401.8	19401.8
N2	1984160.4	-	-	-	-	-	-	-	-
CH3OH	-	-	-	-	-	8774.2	8774.2	37034.8	37034.0
H2S	-	-	-	-	-	-	-	-	-
SO2	-	-	-	-	-	-	-	-	-
CH4	-	-	-	-	-	11832.1	11832.1	12501.6	12500.7
O2	-	-	-	-	-	-	-	-	-
NO2	-	-	-	-	-	-	-	-	-
DME	-	-	-	-	-	-	-	-	-

Stream #	10	11	12	13	14	15	16	17	18
Temperature, °F	199.9	110.0	108.6	255.0	96.9	482.0	462.0	462.0	60.0
Pressure, psia	278.0	272.0	272.0	98.0	14.7	14.2	475.3	475.3	14.7
Mass flow, lb/hr									
CO2	293.0	17337.2	866.9	93.6	960.5	5702.2	-	-	199.4
CO	44.7	28098.0	1404.9	39.6	1444.5	0.0	-	-	5.1
H2	5.1	41924.5	1447.8	5.1	1452.9	0.0	-	-	0.0
H2O	18269.5	1132.4	56.6	17002.2	56.9	16936.1	1454.5	71268.3	1267.3
N2	-	-	-	-	-	71859.5	-	-	-
CH3OH	27798.0	9236.0	461.8	164.7	487.5	-	-	-	27633.3
H2S	-	-	-	-	-	-	-	-	-
SO2	-	-	-	-	-	-	-	-	-
CH4	45.8	12454.8	622.7	34.3	657.1	-	-	-	11.5
O2	-	-	-	-	-	6111.9	-	-	-
NO2	-	-	-	-	-	-	-	-	-
DME	-	-	-	-	-	-	-	-	-

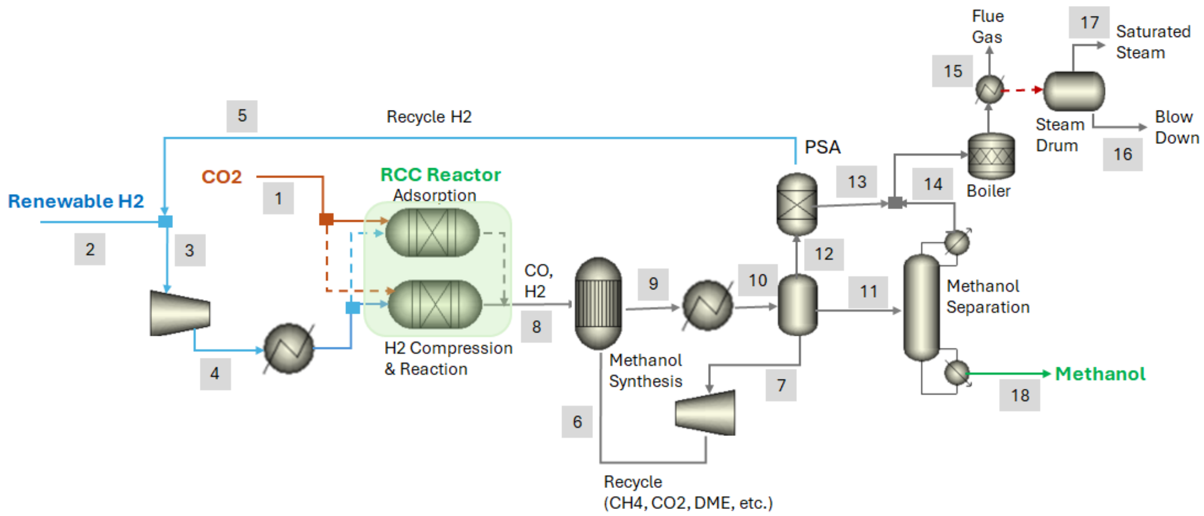

Figure S-2. ASPEN process flow diagrams for MeOH production from RCC-to-CO followed by MeOH synthesis (“Indirect RCC-to-CO”) using K/ZA DFM.

Table S-2. ASPEN model stream table for the “Indirect RCC-to-CO” case (as shown in Figure S-2)

Stream #	1	2	3	4	5	6	7	8	9
Temperature, °F	60.0	60.0	95.2	108.2	110.0	177.9	127.4	482.0	482.0
Pressure, psia	14.7	350.0	350.0	29.4	567.0	731.0	562.0	19.4	580.0
Mass flow, lb/hr									
CO2	220462.3	-	-	-	-	86871.8	86871.8	20723.5	94017.5
CO	-	-	-	-	-	11981.9	11981.9	14520.9	12652.0
H2	-	5988.1	20196.7	20196.7	14208.6	18499.9	18499.9	19050.1	33691.3
H2O	-	-	-	-	-	319.3	319.3	9792.7	15668.3
N2	1984160.4	-	-	-	-	-	-	-	-
CH3OH	-	-	-	-	-	4076.7	4076.7	-	29804.2
H2S	-	-	-	-	-	-	-	-	-
SO2	-	-	-	-	-	-	-	-	-
CH4	-	-	-	-	-	3366.3	3366.3	201.9	3567.9
O2	-	-	-	-	-	-	-	-	-
NO2	-	-	-	-	-	-	-	-	-
DME	-	-	-	-	-	-	-	-	-

Stream #	10	11	12	13	14	15	16	17	18
Temperature, °F	246.1	157.5	110.0	109.2	29.1	482.0	462.0	462.0	59.4
Pressure, psia	575.0	567.0	567.0	567.0	14.7	14.2	475.3	475.3	14.7
Mass flow, lb/hr									
CO2	96591.0	2573.5	91444.0	4572.2	2554.7	9125.2	-	-	18.7
CO	12691.4	39.4	12612.6	630.6	39.4	-	-	-	-
H2	33700.5	9.1	33682.1	973.7	9.1	-	-	-	-
H2O	31000.5	15332.2	336.1	16.8	0.9	10662.4	185.7	9099.3	1266.8
N2	-	-	-	-	-	41756.1	-	-	-
CH3OH	55317.1	25512.9	4291.3	214.6	66.8	0.0	0.0	0.0	25446.1
H2S	-	-	-	-	-	-	-	-	-
SO2	-	-	-	-	-	-	-	-	-
CH4	3592.4	24.5	3543.5	177.2	24.4	-	-	-	-
O2	-	-	-	-	-	-	-	-	-
NO2	-	-	-	-	-	-	-	-	-
DME	-	-	-	-	-	-	-	-	-

The current TEA study involves an nth plant scenario where the technology is considered mature, and the operation will function efficiently, differing from those in the laboratory (i.e., Ref 10 and 11). For the current n^{th} plant operation, the RCC reactor system operates in a cyclic mode between two reactors: one for CO_2 adsorption (R1) and the other for reactive desorption (R2). In Aspen, we assumed both the adsorption and reactive desorption processes take 15 minutes each. Once the products from reactive desorption step are removed from R2, a fresh batch of CO_2 flue gas is quickly introduced into the same reactor, which cools down the reactor, and CO_2 capture takes place, which lasts for 15 minutes. Meanwhile, R1 is pressurized with hydrogen and undergoes reactive desorption for a duration of 15 minutes. And the cycle repeats. We assume the CO_2 adsorption efficiency is 20%, which means 80% of CO2 making contact with the DFM is not adsorbed and is released. Both the RCC-to-methanol and RCC-to-CO reactions are exothermic, producing heat at rates of 22.3 MW and 16.5 MW, respectively. This heat is recovered and integrated into the process. In our model, it is assumed that boiler feed water is cross-exchanged within the reactor to produce steam for the process. Additionally, our model is designed to recover and reintegrate 98% of the heat, after factoring a 2% heat loss from the reactor.

Table S-3. ASPEN model outputs used in TEA calculations. Note: these results have been scaled from their original values in Tables S-1 and S-2 to achieve a constant methanol output across all cases.

Methanol production process	RCC-to-MeOH	RCC-to-CO
Heat exchanger network	$1,267,000	$1,286,000
RCC reactors and ancillary	$4,931,000	$2,306,000
Methanol reactor and ancillary	-	$8,022,000
Pessure swing adsorption	$2,654,000	$3,032,000
Methanol purification and recovery	$1,822,000	$2,537,000
Balance of plant	$1,554,000	$ 4,151,000
Total installed costs	$12,225,000	$21,333,000
Fixed capital investment	$23,017,000	$39,434,000
Capital cost	$1,151,000	$1,972,000
Total capital investment [$]	$24,167,460	$41,404,743
Fixed operating cost [$/t]	$1,325,388	$1,628,894
RCC catalyst used [t]	930	413
MeOH catalyst used [t]	0	54
Electricity used [kWh/hr]	6,718	61,258
Hydrogen used [kg/hr]	3,624	3,270
CO2 used [kg/hr]	110,546	120,407
Indirect CO2 emissions [kg/hr]	465	234
Process water consumption [kg/hr]	36,612	167,513
Methanol yield [kg/hr]	14,600	14,600
Plant capacity factor [%]	90%	90%
Annual methanol production [t/y]	115,104	115,104

<u>TEA Calculations</u>

Details on the TEA methodology have previously presented in the Supporting Information to Martin et al.[1] and will not be repeated in full here. To summarize, a levelized cost (LC) calculation is performed for the methanol system to determine the Levelized Cost of Methanol (LCOM). For some systems, intermediate products are generated to feed the methanol system, and a LC is calculated for these as well: electricity – Levelized Cost of Electricity (LCOE), hydrogen – Levelized Cost of Hydrogen (LCOH), and carbon dioxide – Levelized Cost of CO2 (LCOC). All of these LC calculations use the same equation:

$$LC = (FCR * TASC/TOC * TOC + FOC) / AP + VOC \quad (1)$$

The other terms in equation (1) are:

- TOC: Total overnight cost [$]
- FOC: Fixed operating cost [$/yr]
- VOC: Variable operating cost [$/MWh, for energy OR $/t, for materials]
- AP: Annual production [MWh/yr, for energy OR t/yr, for materials]. The AP for methanol was set to a constant of 115,104 t/y to match previous work, while the AP for electricity, hydrogen, and CO2 was adjusted to achieve this AP for methanol.
- TASC/TOC: Multiplier applied to convert TOC into Total As-Spent Cost (TASC), accounting for escalation/interest on capital over the expenditure period. This study uses 1.093 as the value, reflecting a 3 year expenditure period and 30 year recovery period.[2]
- FCR: Fixed charge rate – the annual percentage of the TASC that must be recovered each year to pay off debts on the capital over the project lifetime. This study uses 7.07% as the value, reflecting a 3 year expenditure period and 30 year recovery period.[2]

Table S-4. Levelized Cost of Methanol (LCOM) calculations using equation (1)

Production Process	Baseline SMR	Baseline CO_2 Hydrogenation	RCC-to-MeOH	RCC-to-CO
TOC value [$]	$100,108,418	$136,000,146	$24,167,460	$41,404,743
TOC source	Woods et al.[3]	See Martin et al. SI[1]	ASPEN Modeling	ASPEN Modeling
FOC value [$/yr]	$2,644,205	$2,653,002	$1,325,388	$1,628,894
FOC source	Woods et al.[3]	See Martin et al. SI[1]	ASPEN Modeling	ASPEN Modeling
VOC value [$/kg]	$0.1400	$0.6100	$0.7516	$0.8012
VOC source	Table S-6	Table S-6	Table S-6	Table S-6
**LCOM [$/kg]**	**$0.230**	**$0.724**	**$0.779**	**$0.843**

Electricity, hydrogen, and CO2, the amount of each input required relative to the methanol output are indicated by the ratios $R_{<component\_name>}$. These are used to calculate LCOE, LCOH, and LCOC using methods previously shown in the SI of Martin et al. SI[1].

Table S-5. Calculating contribution to the Variable operating cost (VOC) from electricity, hydrogen, and carbon dioxide

Methanol Production Process	Baseline SMR	Baseline CO2 Hydrogenation	RCC-to-MeOH	RCC-to-CO
R_{elec} [kWe in / kg MeOH]	-0.338*	0.095	0.461	4.196
R_{elec} source	Woods et al.[3]	Nyari et al.[4]	Table S-3	Table S-3
R_{H2} [kg H_2 in / kg MeOH]	0	0.195	0.248	0.224
R_{H2} source	-	Nyari et al.[4]	Table S-3	Table S-3
RCO_2 [kg CO_2 in / kg MeOH]	0	1.423	7.572	8.247
RCO_2 source	-	Nyari et al.[4]	Table S-3	Table S-3
LCOE [$/kWh]	0.039	0.039	0.039	0.039
LCOH [$/kg H_2]	-	2.637	2.585	2.608
LCOC [$/kg CO_2]	-	0.140	0.000**	0.000**
VOC_{elec} [$/kg MeOH]	-0.009	0.004	0.018	0.164
VOC_{H2} [$/kg MeOH]	0.000	0.514	0.642	0.584
VOC_{CO2} [$/kg MeOH]	0.000	0.078	0.000	0.000

* The Baseline SMR plant is a net electricity generator and sells its excess electricity to the grid.
** There is no cost of CO2 for the RCC plants, since they are adsorbing CO2 from free flue gas.

These VOC components were combined with other process inputs and credits listed in Table S-6. Natural Gas cost was based on a price of $4/MMBTU needed for process heating.

Table S-6. Calculating total Variable operating cost (VOC) from all components

VOC Component All units [$/kg MeOH]	Baseline SMR	Baseline CO2 Hydrogenation	RCC-to-MeOH	RCC-to-CO
Electricity (VOC_{elec})	-0.0093	0.0037	0.0180	0.1645
Hydrogen (VOC_{H2})	0.0000	0.5143	0.6418	0.5843
Carbon Dioxide (VOC_{CO2})	0.0000	0.0781	0.0000	0.0000
Natural Gas	0.1370	0.0131	0.0000	0.0000
Methanol Synthesis Catalyst	0.0002	0.0008	0.0000	0.0046
Autothermal Reformer Catalyst	0.0010	0.0000	0.0000	0.0000
RCC Catalyst Base	0.0000	0.0000	0.0967	0.0338
RCC Catalyst IWI	0.0000	0.0000	0.0246	0.0109
RCC Reactor Steam Credits + Other Methanol Reactor Costs	0.0111	0.0000	-0.0295	0.0031
**Total VOC**	0.1400	0.6100	0.7516	0.8012

References

(1) Martin, J. A.; Tan, E. C.; Ruddy, D. A.; King, J.; To, A. T. Temperature–Pressure Swing Process for Reactive Carbon Capture and Conversion to Methanol: Techno-Economic Analysis and Life Cycle Assessment. *Environ. Sci. Technol.* **2024**, *58* (31), 13737-13747, DOI: 10.1021/acs.est.4c02589

(2) Theis, J.. *Cost Estimation Methodology for NETL Assessments of Power Plant Performance*; DOE/NETL-PUB-22580; U.S. Department of Energy, National Energy Technology Laboratory, 2021. DOI: 10.2172/1567736.

(3) Woods, M.; Kuehn, N.; Shah, V.; White lll, C. W.; Goellner, J. F. *Baseline Analysis of Crude Methanol Production from Coal and Natural Gas*; DOE/NETL-341/101514; U.S. Department of Energy, National Energy Technology Laboratory, 2014. DOI: 10.2172/1601964.

(4) Nyári, J.; Magdeldin, M.; Larmi, M.; Järvinen, M.; Santasalo-Aarnio, A. Techno-economic barriers of an industrial-scale methanol CCU-plant. *J. CO2 Util.* **2020**, *39*, 101166, DOI: 10.1016/j.jcou.2020.101166
